# SARS‐CoV‐2 RNA load in nasopharyngeal specimens from outpatients with breakthrough COVID‐19 due to Omicron BA.1 and BA.2

**DOI:** 10.1002/jmv.28079

**Published:** 2022-08-29

**Authors:** Paula de Michelena, Beatriz Olea, Ignacio Torres, Fernando González‐Candelas, David Navarro

**Affiliations:** ^1^ Microbiology Service, Clinic University Hospital INCLIVA Health Research Institute Valencia Spain; ^2^ Joint Research Unit Infection and Public Health FISABIO‐University of Valencia Institute for Integrative Systems Biology (I2SysBio, UV‐CSIC) and CIBER in Epidemiology and Public Health Valencia Spain; ^3^ Department of Microbiology, School of Medicine University of Valencia Valencia Spain

**Keywords:** BA.1, BA.2, COVID‐19, Omicron variant, SARS‐CoV‐2, vaccine, viral load

## Abstract

This retrospective observational study compared severe acute respiratory syndrome coronavirus 2 (SARS‐CoV‐2) RNA load in nasopharyngeal specimens (NPs) from patients with breakthrough coronavirus disease 2019 (COVID‐19) caused by the Omicron BA.1 or BA.2 sublineages. The convenience sample was composed of 277 outpatients (176 female/112 male; median age, 48 years; range, 12–97) with breakthrough COVID‐19 (*n* = 130 due to BA.1 and *n* = 147 due to BA.2). All participants had completed a full vaccination schedule and 56% had received a booster vaccine dose at the time of COVID‐19 breakthrough microbiological diagnosis. NPs were collected within 7 days (median 2 days) after symptom onset. The TaqPath COVID‐19 Combo Kit (Thermo Fisher Scientific) was used to estimate viral loads in NPs. Overall, viral RNA loads in NPs were comparable (*p* = 0.31) for BA.1 (median, 7.1 log_10_ copies/ml; range, 2.7–10.6) and BA.2 (median, 7.5 log_10_ copies/ml; range, 2.7–10.6), yet peak viral load appeared to be reached sooner for BA.2 than for BA.1 (Day 1 vs. Days 3–5; *p* = 0.002). Time elapsed since last vaccine dose had no significant impact on SARS‐CoV‐2 RNA loads in the upper respiratory tract (URT) for either BA.1 or BA.2. The data presented do not support that the transmissibility advantage of BA.2 over BA.1 is related to generation of higher viral loads in the URT early after infection.

## 1 INTRODUCTION

The severe acute respiratory syndrome coronavirus 2 (SARS‐CoV‐2) Omicron variant currently comprises five sublineages known as BA.1, BA.2, BA.3, BA.4, and BA.5.^1^ February 2022 saw a rapid surge of Omicron BA.2 in Spain. Since then, this subvariant has outcompeted the original Omicron BA.1 to become overtly dominant by May 2022.^2^ Global surveillance data strongly suggest an enhanced transmissibility of BA.2 compared with BA.1,^3^, ^4^ yet the reasons for current BA.2 dominance remain to be firmly established. Data have shown that all Omicron sublineages including BA.2 are comparably neutralized by Omicron patient sera,^5^ and that similar neutralizing antibody titers against BA.2 and BA.1 are elicited in messenger RNA (mRNA) vaccine‐boosted individuals.^6^ This supports the notion that rapid spread of BA.2 is related to increased transmissibility rather than enhanced immunologic escape, which fits to a susceptible infectious and recovered‐type model.^7^ Increased viral load in the upper respiratory tract (URT) upon BA.2 breakthrough infection may partly explain its increased transmissibility compared with BA.1; nevertheless, the few studies addressing this issue have returned conflicting results.^8^, ^9^, ^10^, ^11^ Here we compared SARS‐CoV‐2 RNA load in the URT in fully vaccinated patients with breakthrough coronavirus disease 2019 (COVID‐19) caused by either Omicron BA.1 or BA.2.

## 2 MATERIAL AND METHODS

### 2.1 Patients

In this retrospective observational study, we recruited a convenience sample of 277 outpatients (176 female/112 male) with breakthrough COVID‐19 (*n* = 130 due to BA.1 and *n* = 147 due to BA.2), with a median age of 48 years (range, 12–97) and attended at the Clínico‐Malvarrosa Health Department between February 1 and March 23, 2022. The study was approved by the INCLIVA Research Ethics Committee and informed consent was waived due to its retrospective nature.

### 2.2 Reverse transcription polymerase chain reaction (RT‐PCR) assay

SARS‐CoV‐2 RNA loads in nasopharyngeal specimens (NPs) were estimated using the TaqPath COVID‐19 Combo Kit (Thermo Fisher Scientific) calibrated to the AMPLIRUN® TOTAL SARS‐CoV‐2 RNA Control (Vircell SA) as previously reported.^12^ The Omicron subvariants were identified based on TaqPath S gene target failure (SGTF) and non‐SGTF detection profiles for BA.1 and BA.2, respectively. This discriminative criterion proved 100% accurate by whole‐genome sequencing (not shown) in 149 specimens (BA.1, *n* = 101; BA.2, *n* = 48).

### 2.3 Statistical analysis

Medians of continuous variables were compared by Mann–Whitney *U* test or Kruskal–Wallis test, as appropriate. Frequency comparison across groups was performed using Fisher's exact test. Two‐sided exact *p* were reported. A *p* < 0.05 was considered statistically significant. The analyses were performed using SPSS version 20.0 (SPSS).

## 3 RESULTS

**Figure 1:**
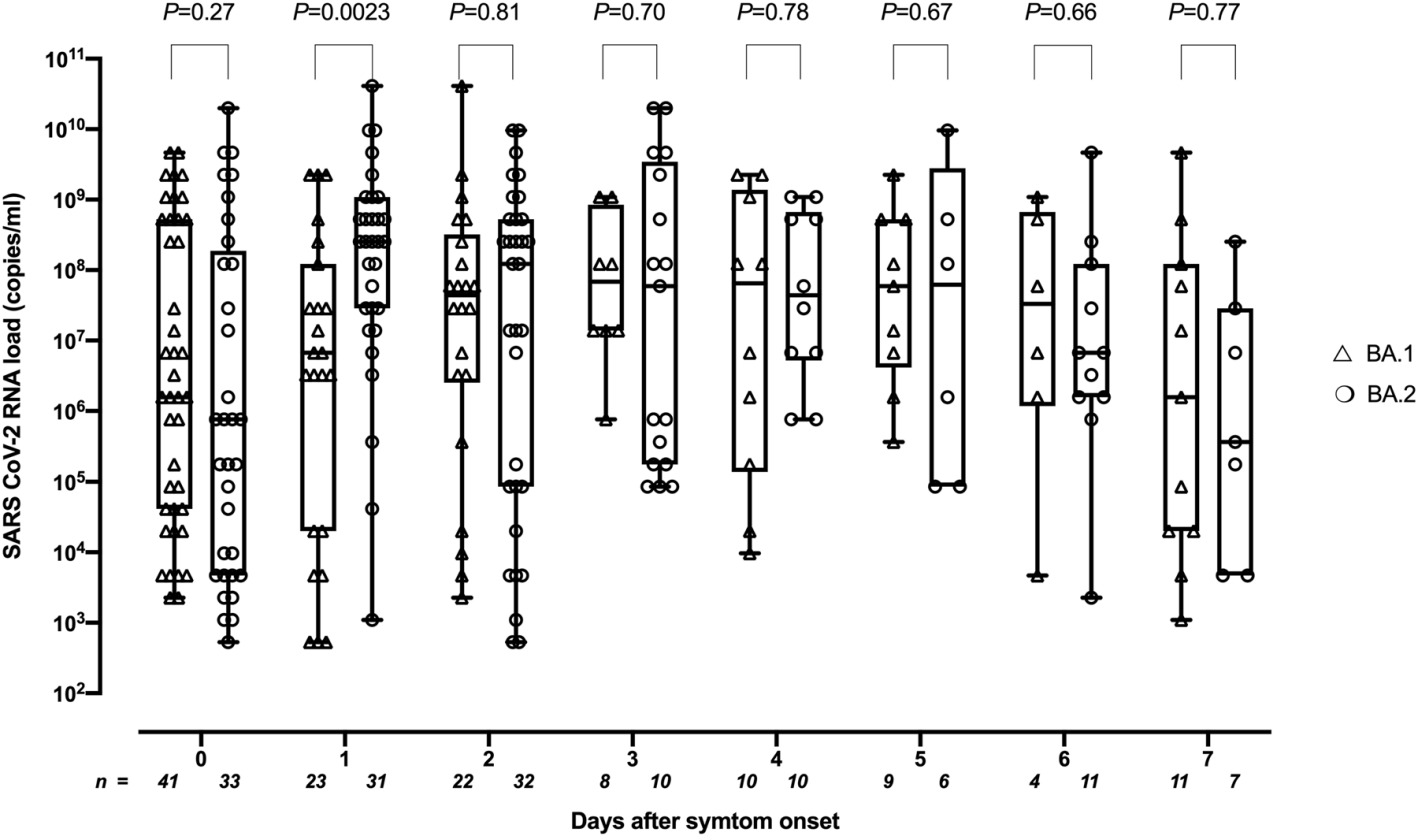
Box–whisker plots depicting severe acute respiratory syndrome coronavirus 2 (SARS‐CoV‐2) RNA loads in nasopharyngeal specimens (NPs) from patients with breakthrough coronavirus disease 2019 (COVID‐19) caused by either the Omicron BA.1 or BA.2 sublineages according to time elapsed since symptom onset. *p* values for comparisons are shown.

**Figure 2:**
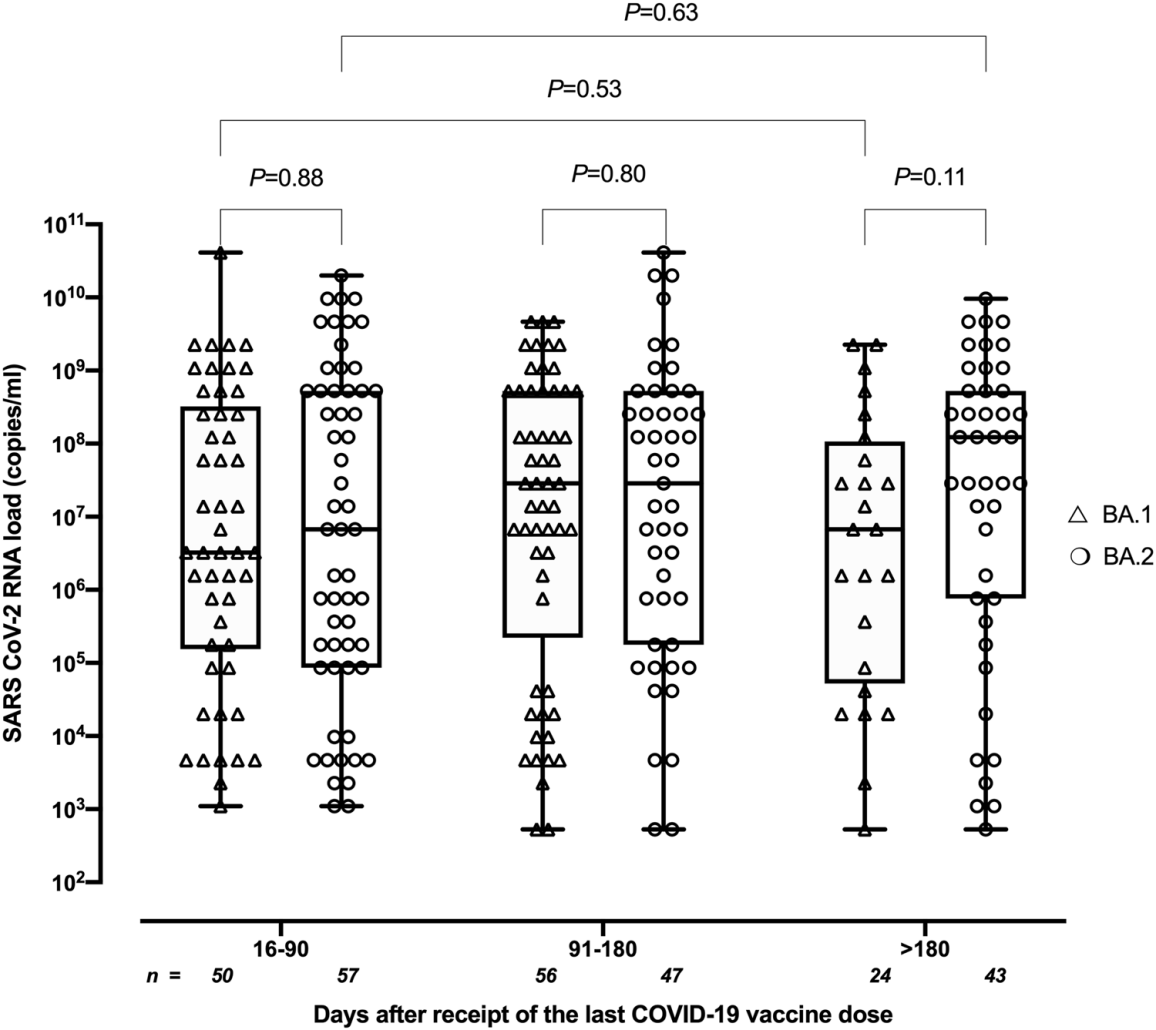
Box–whisker plots depicting severe acute respiratory syndrome coronavirus 2 (SARS‐CoV‐2) RNA loads in nasopharyngeal specimens (NPs) from patients with breakthrough coronavirus disease 2019 (COVID‐19) caused by either the Omicron BA.1 or BA.2 sublineages according to time elapsed since receipt of last vaccine dose. *p* values for comparisons are shown.

A total of 277 COVID‐19 patients were included. Most presented with paucisymptomatic disease and only 28 eventually required hospitalization (24 due to BA.1 and 4 to BA.2; *p* = 0.007). All patients had completed a COVID‐19 vaccination schedule (in most cases mRNA‐based vaccines) and around 60% had been boosted with heterologous or homologous vaccines. As shown in Table 1, patients with COVID‐19 due to BA.1 (*n* = 130) and BA.2 (*n* = 147) were matched for age, sex, SARS‐CoV‐2 vaccination status, time since receipt of the last vaccine dose (median, 109 days vs. 104 days), and time of RT‐PCR testing after symptom onset (median 2 days, range, 0–7 days). It is noteworthy that cellularity in NP specimens from BA.1‐ and BA.2‐infected individuals was comparable, as shown by human β‐glucuronidase gene‐RT‐PCR‐based assay^13^ (not shown). Overall, viral RNA loads in NPs were comparable (*p* = 0.31) for BA.1 (median, 7.1 log_10_ copies/ml; range, 2.7–10.6) and BA.2 (median, 7.5 log_10_ copies/ml; range, 2.7–10.6). When SARS‐CoV‐2 RNA loads from participants were plotted against time elapsed since symptoms onset (Figure 1), peak viral load appeared to be reached sooner for BA.2 than for BA.1 (Day 1 vs. Day 3–5; *p* = 0.002). However, remarkable individual variability was noted in both comparison groups. As time elapsed since vaccination appears to exert a major impact on the magnitude of SARS‐CoV‐2 RNA load in NP collected from COVID‐19 patients with Delta variant COVID‐19,^13^ we assessed whether this could be extended to Omicron BA.1 and BA.2. In line with in the above‐mentioned study,^13^ we arbitrarily set three time frames for comparisons (Days 16–90, 91–180, and >180). As shown in Figure 2, time elapsed since receipt of last vaccine dose had no significant impact on SARS‐CoV‐2 RNA loads in the URT for either BA.1 or BA.2, although a clear trend towards higher viral loads with time since vaccination was evident for BA.2 but not for BA.1. Moreover, SARS‐CoV‐2 RNA load did not differ in magnitude across BA.1 and BA.2 at any given period.

**Table 1:** Relevant features of patients with breakthrough COVID‐19 either due to SARS‐CoV‐2 Omicron BA.1 or BA.2 lineages

Parameter	SARS‐CoV‐2 Omicron variant lineage causing breakthrough COVID‐19		*p*
	BA.1 (*n* = 130)	BA.2 (*n* = 147)	
Age in years (median/range)	48/12‐97	49/13‐97	0.48
Sex (female/male)	84/50	92/64	0.58
RT‐PCR testing after symptoms onset in days (median/range)	2/0‐7	2/0‐7	0.47
No. of patients who completed a regular vaccination schedule (type)	59 (Comirnaty®, *n* = 48; Spikevax®, *n* = 10; Vaxzevria®, *n* = 1)	62 (Comirnaty®, *n* = 50; Spikevax®, *n* = 9; Vaxzevria®, *n* = 3)	0.59
No. of patients who received a booster vaccine dose (type)	71 (Homologous, *n* = 38/Heterologous, *n* = 33)	85 (Homologous, *n* = 38; Heterologous, *n* = 47)	0.61
Day since receipt of the last vaccine dose (median/range)	109/20‐434	104/19‐326	0.66
No. of patients with a record of SARS‐CoV‐2 infection previous to vaccination	7	17	0.08

Abbreviations: RT‐PCR, reverse transcription polymerase chain reaction; SARS‐CoV‐2, severe acute respiratory syndrome coronavirus 2.

## 4 DISCUSSION

The data presented herein suggested that overall SARS‐CoV‐2 Omicron RNA loads in NPs do not differ significantly across BA.1 and BA.2 in patients with breakthrough COVID‐19 sampled within 7 days (median, 2 days) since symptom onset. Participants in the current study had all been fully vaccinated, and more than half had received a booster vaccine dose; moreover, few of them (*n* = 24) seemingly exhibited hybrid immunity at the time of testing. This is important given that hybrid immunity, resulting from natural infection and vaccination, may confer increased protection against SARS‐CoV‐2 infection and severe COVID‐19 from different variants of concern including Omicron.^14^, ^15^, ^16^ Whether our observations can be extrapolated to other populations substantially differing in baseline immune response parameters at the time of breakthrough Omicron infection is uncertain. In agreement with our results, Marking et al.^8^ investigated breakthrough infections in triple‐vaccinated healthcare workers with and without prior SARS‐CoV‐2 infection, reporting comparable viral RNA loads in URT for BA.1 and BA.2. In contrast, Qassim et al.^10^ found significantly higher viral RNA loads in BA.2 infections compared with those due to BA.1 (3.5 fewer RT‐PCR C_T_). Nevertheless, age distribution, participant vaccination status, and percentage of individuals with prior SARS‐CoV‐2 infection differed between their study and ours. Likewise, data from a large Swedish study seemingly indicated that Omicron BA.2 infection is associated with ~100‐fold higher viral RNA levels in the URT than BA.1, early in the course of infection.^9^ These differences were not however significant (*p* = 0.06). The lack of information on participant characteristics in their study, in particular regarding their vaccination status and clinical presentation of SARS‐CoV‐2 infection at the time of testing, precludes direct comparison with our study. In our experience, viral RNA load COVID‐19 cases appeared to reach a peak earlier in BA.2 than in BA.1, although this observation should be taken with caution due to the population approach of our analysis. Data from a recent study^11^ suggesting comparable replication kinetics of BA.1 and BA.2 sublineages in cell culture and after SARS‐CoV‐2 Omicron natural infection do not support our findings, yet another study^17^ found Omicron BA.2 to be more fusogenic than BA.1, which may conceivably have an impact on virus replication dynamics in the URT. We speculate that differences in the features of innate immune responses mediated by interferons or by functional antibodies and T cells elicited by BA.2 and BA.1 may account for our findings.

We previously reported in SARS‐CoV‐2‐naïve individuals with breakthrough COVID‐19 that SARS‐CoV‐2 Delta RNA loads in NP increased in parallel to the time elapsed since last vaccine dose. This may also be the case for the Omicron variant according to a recent study.^10^ Here we found a trend for a direct relationship between time elapsed since last vaccine dose and magnitude of SARS‐CoV‐2 load in NP for BA.2 but not BA.1. This observation is compatible with a less efficient, more rapidly waning cross‐protective antibody response elicited by Wuhan‐Hu‐1‐based vaccines against BA.2 compared with BA.1, or both.^17^

The current study has several limitations. First, no sequential specimens were available from participants, which impeded precise characterization of SARS‐CoV‐2 RNA load kinetics on the URT at the individual level. Second, no viral cultures were performed to evaluate the content of viable viral particles in NP from patients in the two groups. Third, no data on virus‐specific antibody or T‐cell immunity was available from participants. Fourth, it remains to be determined whether our observations can be extrapolated to hospitalized patients, which were underrepresented in the current series.

In conclusion, the theory that the Omicron BA.2 subvariant is better adapted for replication in the URT tract than BA.1, thus generating higher viral loads in the setting of breakthrough COVID‐19 developing in a highly vaccine‐boosted population, is not supported by the data herein.

## AUTHOR CONTRIBUTIONS

**Paula de Michelena, Ignacio Torres, Beatriz Olea, Ignacio Torres, Fernando González‐Candelas**: methodology and data validation. **David Navarro**: conceptualization, data analysis, and manuscript writing.

## CONFLICT OF INTEREST

The authors declare no conflict of interest.

## Data Availability

The data presented in the manuscript have not been made available, but can be shared upon request.
